# Isoprenyl carboxyl methyltransferase inhibitors: a brief review including recent patents

**DOI:** 10.1007/s00726-017-2454-x

**Published:** 2017-06-19

**Authors:** Woo Seok Yang, Seung-Gu Yeo, Sungjae Yang, Kyung-Hee Kim, Byong Chul Yoo, Jae Youl Cho

**Affiliations:** 1 Department of Genetic Engineering, Sungkyunkwan University, 2066 Seobu-ro, Jang-gu, Suwon, 16419 Republic of Korea; 2 Department of Radiation Oncology, Soonchunhyang University College of Medicine, Soonchunhyang University Hospital, Cheonan, 31151 Republic of Korea; 3 Biomarker Branch, Research Institute, National Cancer Center, Goyang, 10408 Republic of Korea

**Keywords:** ICMT inhibitor, Cancer, Methylation, Cysmethynil, Ras

## Abstract

Among the enzymes involved in the post-translational modification of Ras, isoprenyl carboxyl methyltransferase (ICMT) has been explored by a number of researchers as a significant enzyme controlling the activation of Ras. Indeed, inhibition of ICMT exhibited promising anti-cancer activity against various cancer cell lines. This paper reviews patents and research articles published between 2009 and 2016 that reported inhibitors of ICMT as potential chemotherapeutic agents targeting Ras-induced growth factor signaling. Since ICMT inhibitors can modulate Ras signaling pathway, it might be possible to develop a new class of anti-cancer drugs targeting Ras-related cancers. Researchers have discovered indole-based small-molecular ICMT inhibitors through high-throughput screening. Researchers at Duke University identified a prototypical inhibitor, cysmethynil. At Singapore University, Ramanujulu and his colleagues patented more potent compounds by optimizing cysmethynil. In addition, Rodriguez and Stevenson at Universidad Complutense De Madrid and Cancer Therapeutics CRC PTY Ltd., respectively, have developed inhibitors based on formulas other than the indole base. However, further optimization of chemicals targeted to functional groups is needed to improve the characteristics of ICMT inhibitors related to their application as drugs, such as solubility, effectiveness, and safety, to facilitate clinical use.

## Introduction

**Fig. 1:**
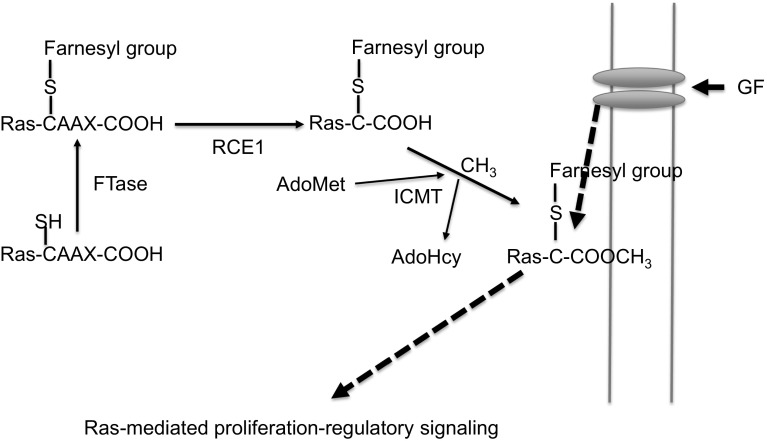
Schematic diagram of the RAS/ICMT regulatory process in a growth factor-inducing signaling cascade. *FTase* farnesyltransferase, *RCE1* Ras-converting CAAX endopeptidase 1, *ICMT* isoprenylcysteine methyltransferase, *AdoMet S*-adenosyl-l-methionine, *AdoHcy S*-adenosyl-l-homocysteine, *GF* growth factor

The post-translational modification of Ras family members is a crucial step in inducing translocation of Ras proteins from the endoplasmic reticulum to the plasma membrane for management of cell proliferation triggered by growth factor signaling. Mutations in Ras family proteins (e.g., K-RAS) are also closely related to the incidence of various types of cancers in humans, such as pancreatic cancer (Downward 2003; Pylayeva-Gupta et al. 2011). Normally, a series of post-translational modifications of Ras proteins are a prerequisite for localization of Ras in the plasma membrane; however, mutations in Ras proteins can induce uncontrolled activation of Ras signaling pathways and Ras-mediated oncogenesis in the cell membrane. The post-translational modification of Ras first requires farnesylation or geranylgeranylation of the C-terminal CAAX motif, in which covalent bonding of farnesyl or geranylgeranyl isoprenoid lipids to the cysteine residue of CAAX is mediated by farnesyl transferase (FTase) or geranylgeranyl transferase (GGTase I), respectively, as summarized in Fig. 1 (Winter-Vann and Casey 2005; Clarke 1988). During this step, the CAAX endoprotease RCE1 cleaves the three C-terminal amino acids (–AAX) (Ashby 1998), and isoprenyl carboxyl methyltransferase (ICMT) methylates a prenylated cysteine (Bergo et al. 2000). Isoprenyl carboxyl methylated proteins anchor to the cell membrane (Eisenberg et al. 2013) and influence cell signaling pathways. (Bergo et al. 2004; Goodman et al. 1990). ICMT (MW 32 kDa) is essential for post-translational modification of Ras proteins and is localized in the endoplasmic reticulum (Zhang and Casey 1996). Since Ras plays an important role in cancerous signaling pathways, ICMT is also a substantial focus of studies on anti-cancer agents (Wahlstrom et al. 2008). Suppression of the carcinogenic transformation of RAS by inhibition of the enzymes involved in farnesylation or geranylgeranylation has been studied for treatment of cancer. There are several inhibitors of FTase, including lonafarnib (IC_50_ = 1.9 nM) and tipifarnib (IC_50_ = 7.9 nM) (Basso et al. 2006). Although some inhibitors of FTase have shown inhibitory activities in mouse models, they had no notable effect on the clinical score in cancer patients. Moreover, the geranylgeranylated Ras proteins maintain biological activities when FTase is blocked by inhibitors, limiting the effects of those inhibitors (Anderson et al. 2005; Doll et al. 2004; Mazieres et al. 2004; Lene 2009). Numerous reports have also demonstrated that inhibition of ICMT leads to an anti-proliferative effect in cancer (Clarke 1988; Bos 1989; Bishop et al. 2003; Gibbs et al. 1994). Pharmacologic or genetic inactivation of ICMT resulted in cell cycle arrest and apoptosis (Bergo et al. 2000, 2004). Therefore, the strategy of suppressing ICMT with chemical inhibitors could be considered an alternative therapeutic approach targeting Ras-mediated tumorigenic responses (Teh et al. 2014).

## ICMT inhibitor classes

Isoprenyl carboxyl methyltransferase inhibitors are divided into three classes based on their properties. The first class of these inhibitors includes *S*-adenosyl-l-homocysteine (AdoHcy) and its precursors. During methylation, *S*-adenosyl-l-methionine (AdoMet) donates a methyl group to the substrate proteins and is converted to AdoHcy (Pylayeva-Gupta et al. 2011; Anderson et al. 2005; Kim et al. 2013; Perez-Sala et al. 1992), a by-product that acts in a negative feedback reaction and leads to reduced enzyme activity of methyltransferase until it is cleaved by AdoHcy hydrolase (Shi and Rando 1992; Kramer et al. 2003). Therefore, AdoHcy suppresses the activity of ICMT by acting as a non-selective inhibitor of other methyltransferases (Winter-Vann et al. 2003), and specific inhibitors of ICMT are still needed for treatment. *N*-Acetyl-*S*-farnesyl-l-cysteine (AFC) and *N*-acetyl-*S*-geranylgeranyl-l-cysteine (AGGC) can competitively bind to the same substrate tunnel of farnesylated proteins. These structural analogs of prenylcysteine represent the second class of ICMT inhibitors by acting as actual substrates that can compete with Ras (Henriksen et al. 2005). In fact, there are three structural mimics of AFC. The first analog of AFC, which has a sulphonamide linkage in place of an amide bond, had an IC_50_ value of 8.8 μM in a vapor diffusion assay (Hrycyna and Clarke 1990). The other analog of AFC has a triazole moiety in place of an allylic thioether, which can be easily degraded by enzymatic and chemical processes. This analog has an IC_50_ value of 19.4 μM. The third structural mimic possesses an aryl alkyl moiety in the farnesyl side chain of AFC instead of two isoprenoid units and has an IC_50_ value of 34.6 μM (Ramanujulu et al. 2013; Buchanan et al. 2007, 2008a, b). The third class of ICMT inhibitors includes molecules that have a low molecular weight (<900 Da) and are utilized as drugs due to their rapid diffusion through the cell membrane (Macielag 2012). Cysmethynil (2-[5-(3-methylphenyl)-l-octyl-lH-indolo-3-yl]acetamide), which was identified from 10,000 compounds in the chemical library, is a representative small-molecular inhibitor that competes with isoprenylated cysteine of other CAAX-containing proteins, but not AdoMet (Lene 2009). Cysmethynil induces incorrect localization of Ras and interrupts the signaling pathway of cancer cells. Consequently, cysmethynil causes autophagic cell death through pharmacological inhibition of ICMT (Winter-Vann et al. 2003; Henriksen et al. 2005; Wang et al. 2008; Clarke and Tamanoi 2004; Baron et al. 2007). Because the desired concentration of a drug in circulation is determined by its solubility, this measure is a very important aspect of drug development (Savjani et al. 2012). Cysmethynil is insoluble in water and has high lipophilicity and structural flexibility and is, therefore, considered to be non-applicable for clinical use (Kerns and Di 2010), implying the need for improvement through further structure–activity relationship (SAR) studies. The discovery of an effective ICMT inhibitor is essential for the development of cancer drugs, because Ras is associated with numerous cancer signaling pathways according to in vitro and in vivo tumorigenic models (Lau et al. 2014). We described the current patent literature focusing on inhibitors of ICMT as an attractive biological target.

## Current state of patents for ICMT inhibitors

In 2009, Richard A. Gibbs, Brian S. Henriksen, Christine A. Hrycyna, and Jessica L. Anderson (patent requestor: Purdue Research Foundation) filed for a patent for inhibitors of ICMT for use in treating neoplasms and cancer (Donelson et al. 2009). Gibbs’ laboratory has also published a number of papers on ICMT inhibitors (Bergman et al. 2011a, b; Majmudar et al. 2011, 2012; Donelson et al. 2006). In 2011, Patrick J. Casey, Rudi A. Baron, and Ann M. Winter-Vann at Duke University applied for a patent for ICMT inhibitors with potential anti-cancer activity (Casey et al. 2011). From 2013 to 2014, a patent of small-molecule inhibitors of ICMT was filed by the National University of Singapore (Ramanujulu et al. 2013; Leow et al. 2014). We used Worldwide Intellectual Property Service (WIPS) global, the Korea Intellectual Property Rights Information Service (KIPRIS), the Google patent search system, and the PubMed website to examine patents or papers on ICMT inhibitors.

## Patent evaluation

In section “Patent evaluation”, we present the types of ICMT inhibitors and their chemical structures or enzymatic therapies relating to cancer biological activities. The subsections are organized in chronological order by patent applicant.

### Purdue research foundation

**Fig. 2:**
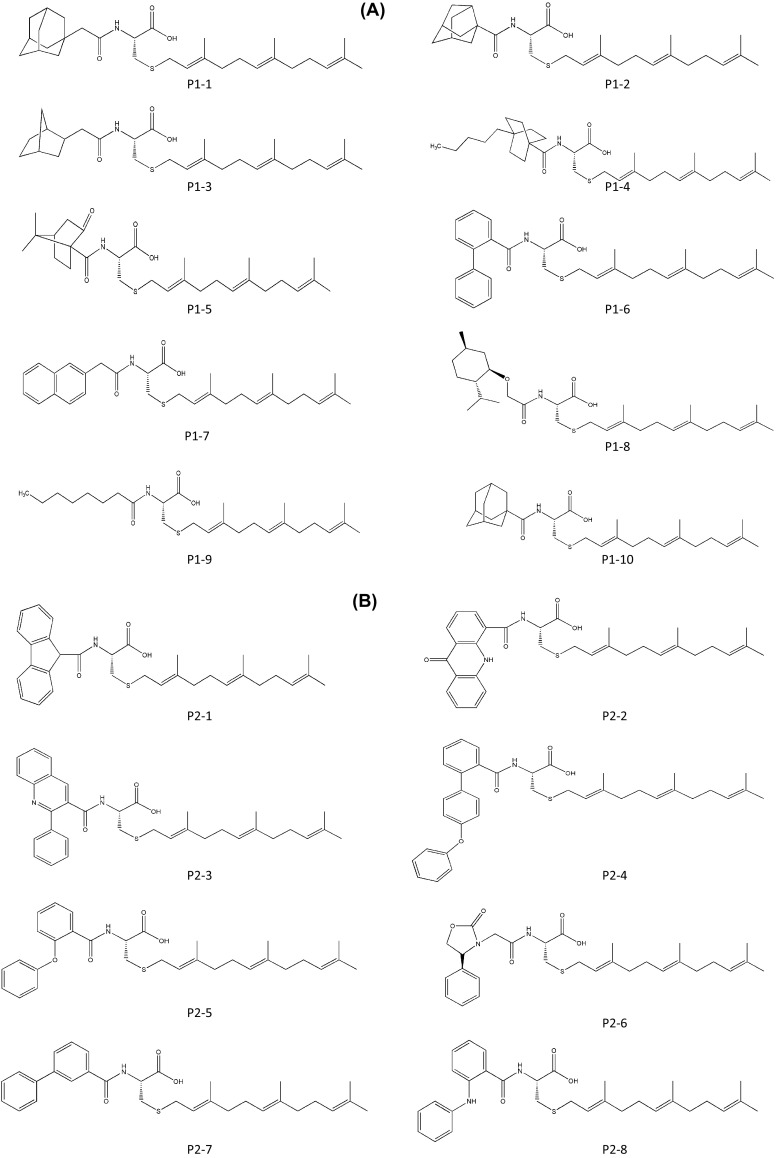

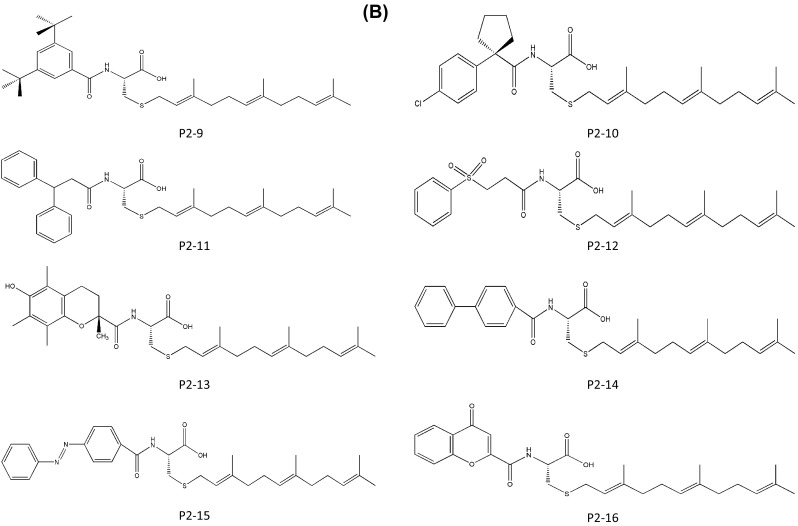
Chemical structures of ICMT inhibitors synthesized by Gibbs (Purdue Research Foundation). **a** Amide-modified farnesyl-cysteine analogs. **b** Subsequent AMFC library

Despite findings that FTase is a key enzyme in the post-translational modification of Ras proteins and other major causative enzymes involved in 30% of human cancers, numerous human tumors associated with mutation of K-Ras are resistant to FTase inhibitors. In the presence of FTase inhibitors, the decreased biological activity of farnesylated K-Ras is compensated for by another form of K-Ras post-translational modification: geranylgeranylation by GGTase I. Therefore, many research centers and institutes have focused on the identification of ICMT inhibitors. At Purdue Research Foundation, Richard A. Gibbs patented ICMT inhibitors generated by solid-phase synthesis of amide-modified farnesyl-cysteine derivatives (AMFCs). His team used the 2-chlorotrityl chloride resin linked to Fmoc-Cys (SStBu)-OH with 1% collidine following connection of a suitable farnesyl side chain to the free thiol using collidine and farnesyl chloride. The Fmoc group was detached by 20% piperidine/DMF, and the selected compound with carboxylic acid was connected to the free amine using HBTU/HOBt. Finally, the resin was removed from the polymer-bound prenylcysteine derivatives by 0.5% TFA (Bonkowski et al. 2013; Merrifield 1963; Dourtoglou et al. 1984). The compounds for the patent containing the library of amide-modified farnesyl-cysteine derivatives were screened by a scintillation proximity assay. Scintillant beads attached to ICMT emit light only when radiolabeled compounds are in proximity and the compounds and ICMT are bound together. Using a spectrophotometer, the degree of light emission is quantitatively recorded and used for selection of candidate ICMT inhibitors (Hertzberg and Pope 2000). Screened compounds resulted in 50% or greater inhibition of ICMT activity at 50 μM. To determine the IC_50_ values of these compounds, the researchers used the vapor diffusion assay with substrate-based analogs derived from several novel scaffolds and *N*-acetyl-*S*-farnesyl-l-cysteine (AFC) as the substrate of ICMT. Results from the work completed under this patent show IC_50_ values ranging from 12.1 to 22.9 μM (compounds P1-1–10; Fig. 2a), based on which compound P1-1 was the best inhibitor of ICMT (IC_50_ values = 12.1 ± 2.1). This compound has been used to synthesize new compounds, because the biphenyl scaffold is more convenient for the generation of derivatives than are adamant scaffolds. The IC_50_ values of the newly synthesized compounds are generally low, ranging from 4.3 to 7.1 μM (compounds P2-1–16; Fig. 2b). Among the analogs, the compound P2-5, *N*-(2-oxyphenyl)benzoyl-l-cysteine[*S*-(2*E*,6*E*)-3,7,11-trimethyldodeca-2,6,10-trienyl]-OH, is three times more potent than the other compounds in the inhibition of ICMT. The enhanced activity of compound P2-5 might be due to its linker element between two phenyl ring structures, which allows greater flexibility in conformational changes, leading to a greater affinity for ICMT.

### Duke University

**Fig. 3:**
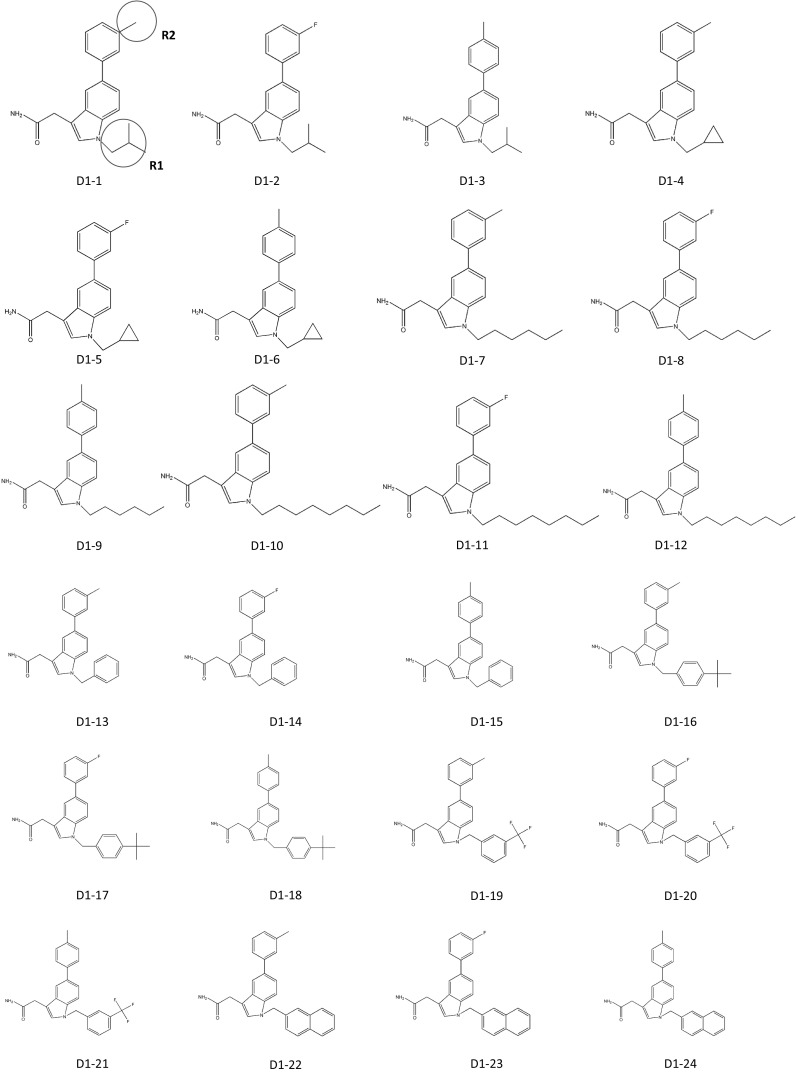

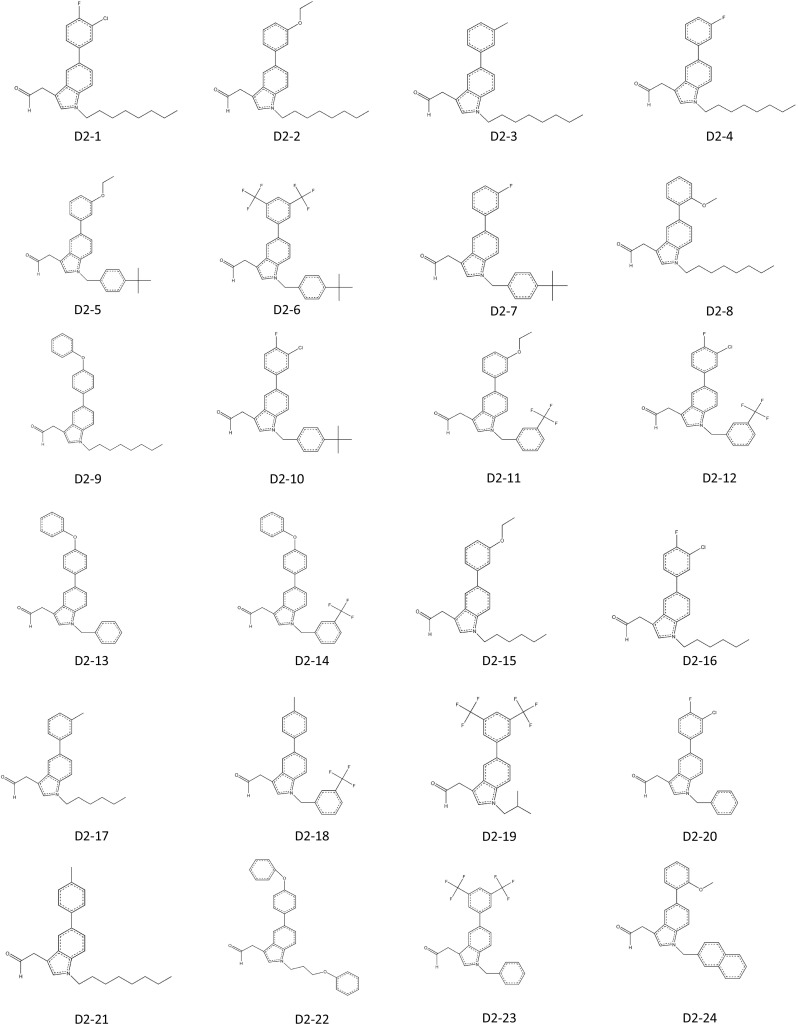

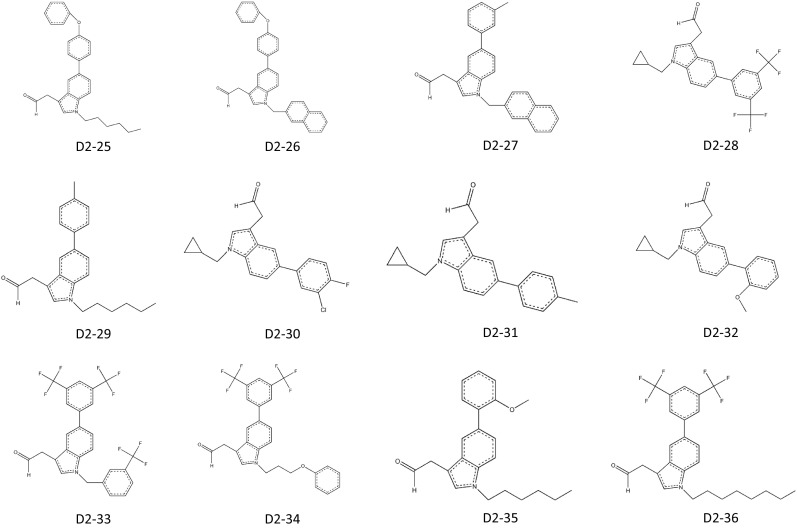
Indole-based small-molecule inhibitors of ICMT developed by Casey (Duke University)

In 2011, Patrick J Casey applied for a patent for indole-based small-molecule inhibitors of ICMT. This invention is based on cysmethynil, a commercially available indole-based inhibitor of ICMT. To test the therapeutic effectiveness of cysmethynil, the inventor measured the enzyme activity of ICMT using biotin-*S*-farnesyl-l-cysteine as a substrate. ICMT activity was decreased by cysmethynil in a time-dependent manner. Cysmethynil inhibited the growth of mouse embryonic fibroblasts and signaling pathways related to cancer cell growth, including MAPK or Akt pathways, in EGF-stimulated conditions and additionally regulated the mislocalization of GFP-tagged Ras (Casey et al. 2011; Winter-Vann et al. 2005). Finally, cysmethynil reduced the steady-state levels of Rho and GTPases. Based on these results, they developed small-molecule inhibitors of ICMT that induced the mislocalization of Ras without inhibition of other enzymes including FTase, Rce1 protease, and AdoMet-dependent DNA methyltransferase. Using the indole-based structure, they substituted various functional groups in the R1 and R2 positions. Their compounds contain three variant R2 substitutions, meta-, para-methyl, and 3-fluoro-phenyl, and various R1 substitutions including isopropyl, cyclopropyl, hexyl, octyl, benzyl, tert-butylbenzyl, trifluoromethyl benzyl, and naphthyl. These compounds displayed IC_50_ values ranging from 2.1 to 19.3 μM (compounds D1-1–24; Fig. 3). The compound D1-10, which contains meta-methyl phenyl in R2 and octyl in R1, showed the best results with an IC_50_ value of 2.1 ± 0.9 μM. To screen for more potent ICMT inhibitors than cysmethynil, they expanded the substitutive variations in both R1 and R2 based on the biphenyl scaffold and obtained IC_50_ values ranging from 1 to <30 μM (compounds D2-1–16; Fig. 3). Among these, compound D2-1 containing octyl in R1 and 3-chloro-4-fluoro-phenyl in R2 showed more effective ICMT inhibitory activity than cysmethynil, with an IC_50_ value for ICMT of 1 μM. This patent demonstrates that cysmethynil exhibits potent and selective inhibition of the enzyme activity of ICMT, and that its efficacy can be further improved by manipulating functional groups.

### National University of Singapore

The National University of Singapore recently received two patents related to small-molecule inhibitors of ICMT with anti-cancer activity. In this section, we introduce the contents of these patents in separate sections.

#### Jo Lene Leow

**Fig. 4:**
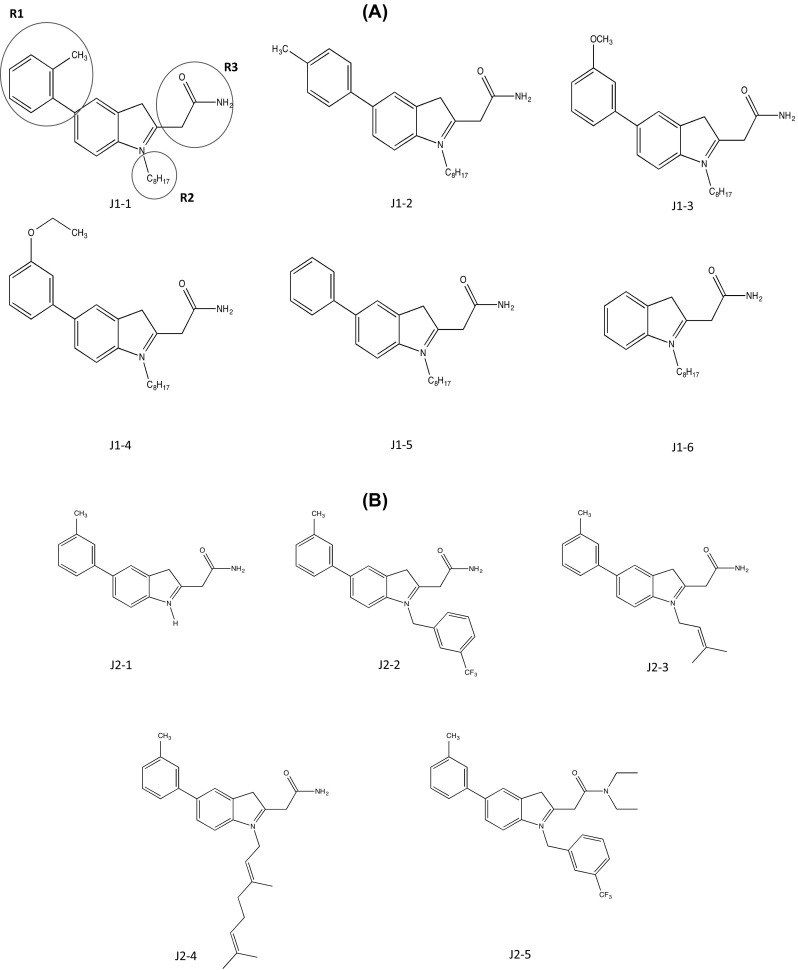

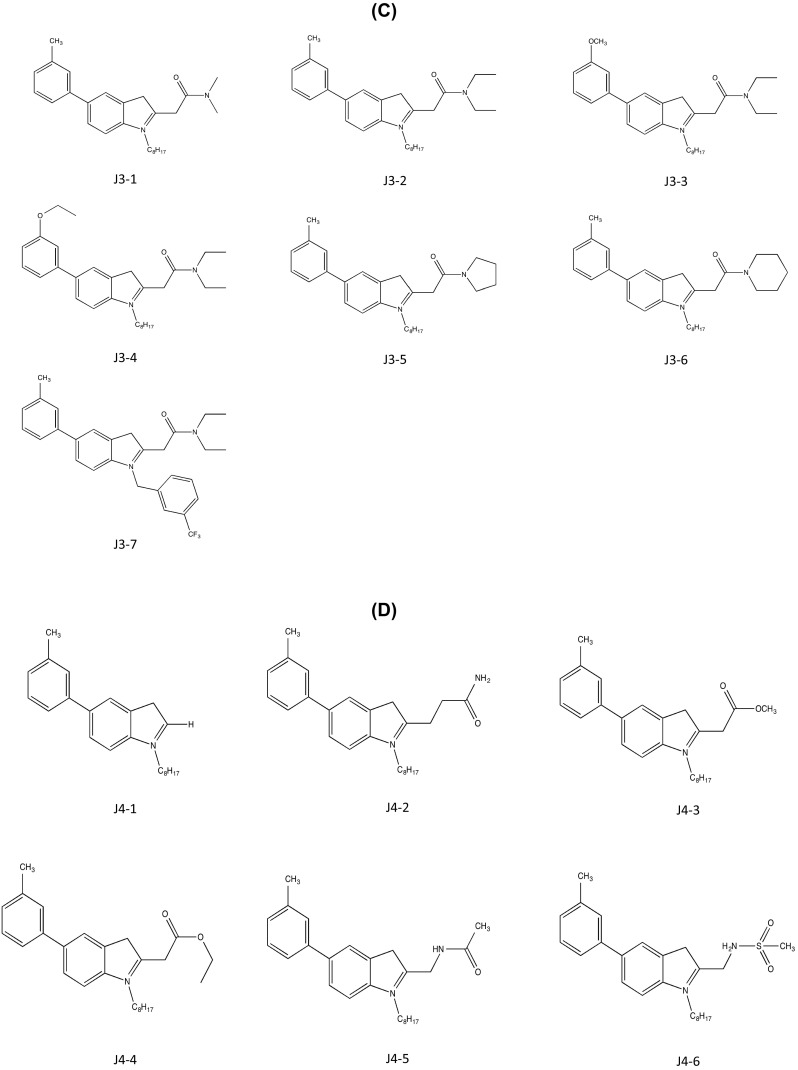

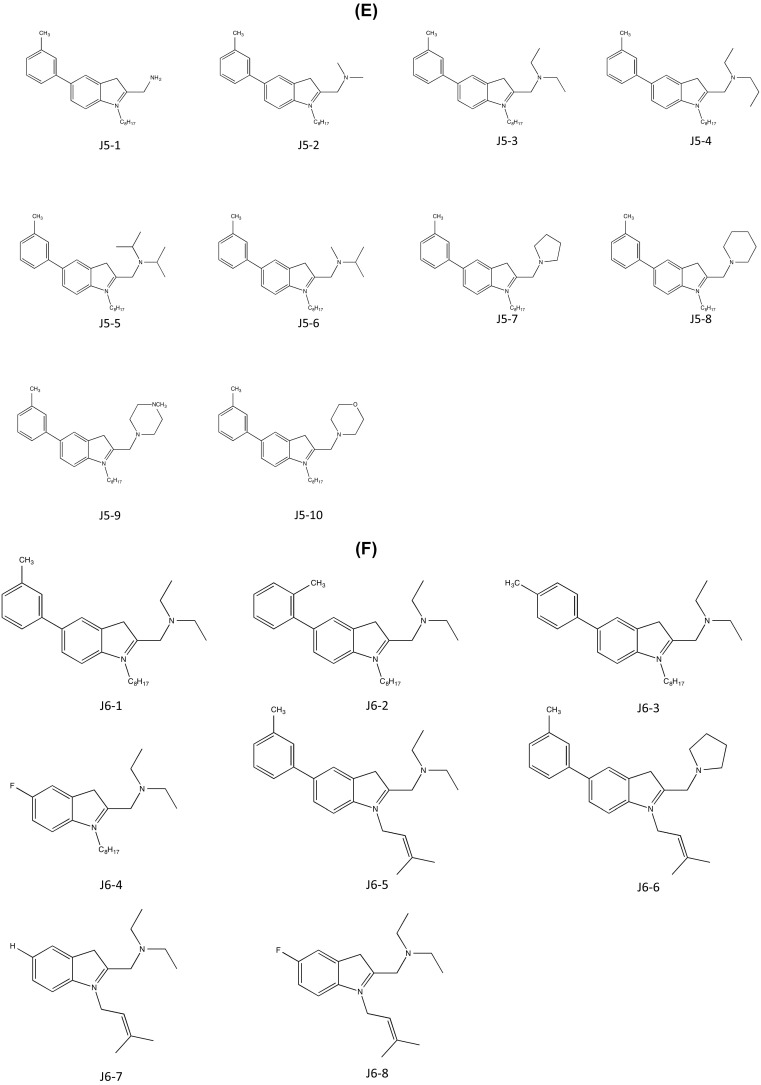
ICMT inhibitors developed by Jo Lene Leow (National University of Singapore). **a** Variation at R1. **b** Variation at R2. **c** Variation at R3-tertiary amides. **d** Variation at R3-other than tertiary amides. **e** Variation at R3-amines. **f** Variation at R1 and R2 with R3=amino

As introduced in section “ICMT inhibitor classes”, cysmethynil is a potential drug candidate for inhibiting ICMT activity. However, the hydrophobicity of cysmethynil is a major obstacle to development of this compound as a drug-related material (Kerns and Di 2010). Therefore, the Jo Lene Leow laboratory synthesized various cysmethynil derivatives with in vitro anti-proliferative activity against MD-MBA231 cells and drug-like properties such as low lipophilicity. Similar to the previous patent (Duke University), synthesized compounds were based on an indole structure. The first series includes fixed substituents at R2 (–*n*-C_8_H_17_) and R3 (–CH_2_CONH_2_) and variations at R1. The ICMT inhibition activity ranged from IC_50_ values of 1.0 to 6.5 μM. Moreover, decreased cell viability was shown with IC_50_ values ranging from 19.1 to <25 μM (Compounds J1-1–6; Fig. 4a). Although compound J1-1 (2-(1-octyl-5-*o*-tolyl-1H-indol-3-yl)acetamide) displayed the best inhibition of ICMT activity with an IC_50_ value of 1.0 μM, the IC_50_ for cell viability of MDA-MB-231 cells was not remarkable. In series 2 with fixed substituents at R1 and R3, variations were introduced at R2, including H-, isoprenyl, geranyl, and trifluoromethyl benzyl. This yielded IC_50_ values of ICMT activity ranging from 1.1 to 33 μM and IC_50_ values for cell viability ranging from 10.6 to 100 μM (compounds J2-1–5; Fig. 4b). In comparison to series 1, the variations at R2 were not suitable for the inhibition of activity and cell viability, although compound J2-4 ((*E*)-2-(1-(3,7-dimethylocta-2,6-dienyl)-5-m-tolyl-1H-indol-3-yl)) acetamide) showed greater inhibitory activity than cysmethynil. In series 3, various tertiary amide substituents are introduced at R3, resulting in ICMT inhibitory activity and cell viability IC_50_ values of 0.8 to 22.9 and 15.6 to >50 μM, respectively (compounds J3-1–7; Fig. 4c). Among these, compound J3-3 (*N*,*N*-diethyl-2-(5-(3-methoxyphenyl)-1-octyl-1H-indol-3-yl)acetamide) showed great inhibition of ICMT activity but had poor effects on cell viability, with IC_50_ values greater than 50 μM. To further investigate the impact of the R3 group on efficiency, they manipulated R3 substituents with groups other than tertiary amides (IC_50_ for ICMT inhibition = 1.2 to >100 μM and IC_50_ for cell viability = 17.1 to >100 μM; compounds J4-1–6; Fig. 4d). The ICMT IC_50_ values of compound J4-2 and compound J4-6 are slightly better than that of cysmethynil; however, both compounds retained the problem of poor solubility seen with cysmethynil. It was speculated that improving the poor solubility of these compounds might further enhance the inhibitory effects. However, the IC_50_ values of ICMT inhibition and cell viability of MDA-MB231 cells were greatly decreased when R3 amides were replaced by less polar amines, thereby rendering the compound lipophilic. IC_50_ values for amines range from 0.5 to 2.7 μM (ICMT inhibition) and 2.9 to 25 μM (cell viability); Log*P* ^3^ values range from 7.6 to 10.6 compared with 7.0 for cysmethynil (compounds J5-1–10; Fig. 4e). Variations in R1 and R2 with R3 fixed as amino groups displayed IC_50_ values for ICMT inhibition and cell viability of cancer cells ranging from 0.6 to 67 and 3.4 to 32 μM, respectively (compounds J6-1–8; Fig. 4f). In contrast to the other analogs, compounds J6-7 and J6-8 (IC_50_ = 67 and 35 μM; Log*P* ^3^ = 5.19 and 5.38) displayed reduced ICMT inhibition and in vitro anti-proliferative activity. The common features between these two compounds are the absence of an aromatic ring in R1 and of an octyl side chain in R2. The compounds J6-4–6 maintained either R1 or R2 from cysmethynil. Since J6-7 and J6-8 were the only analogs in this series with a lower Log*P* ^3^ than cysmethynil, it is reasonable to conclude that the lipophilicity of a compound is not directly linked to the effectiveness of inhibitory activity. The most promising candidate appears to be compound J6-3, with IC_50_ values for inhibition of ICMT and cell viability of 0.6 and 3.4 μM. The anti-proliferative activity of this compound is more than sixfold greater than that of cysmethynil.

#### Pondy Murugappan Ramanujulu Laboratory

**Fig. 5:**
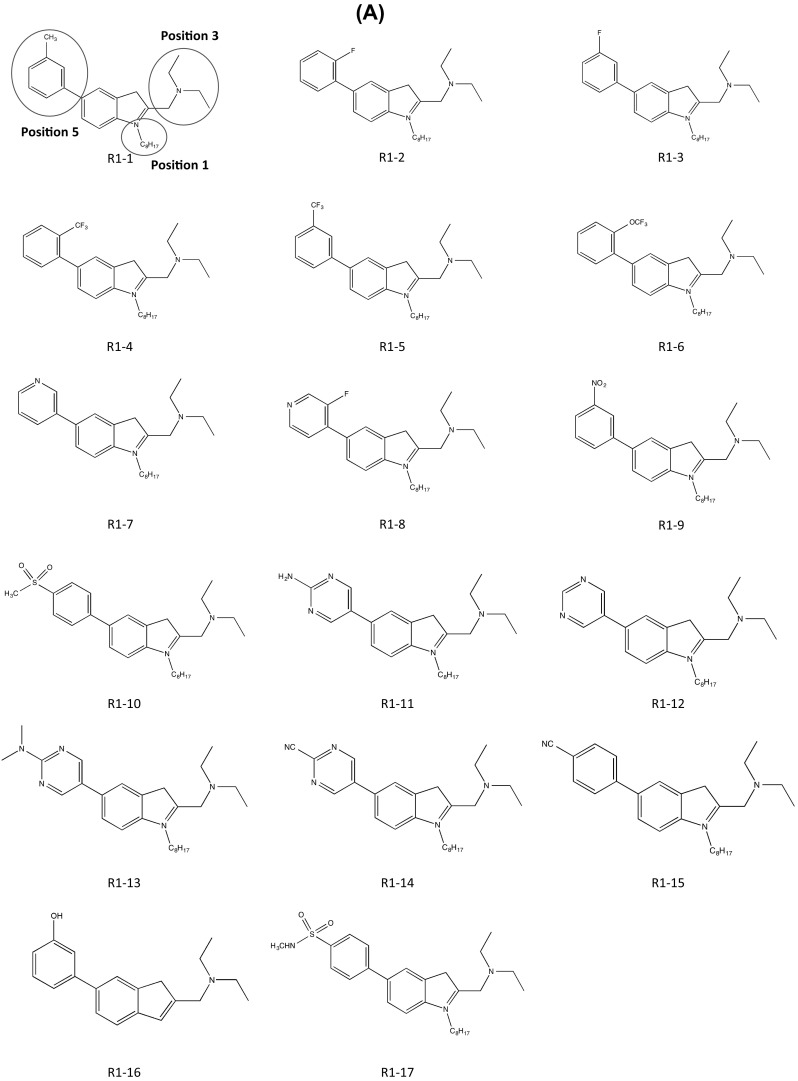

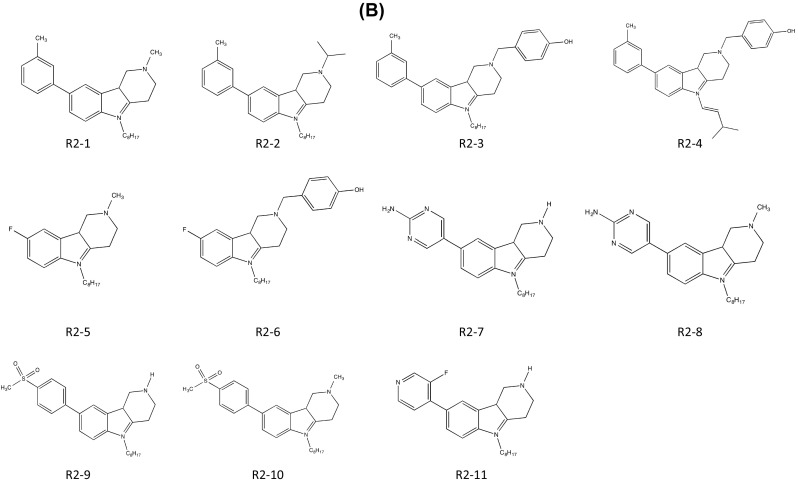
ICMT inhibitors developed by Ramanujulu (National University of Singapore). **a** Small-molecule ICMT inhibitors based on indole. **b** Small-molecule ICMT inhibitors based on tetrahydrocarbolines

Pondy Murugappan Ramanujulu’s team also focused on the hydrophobic chemical structure of cysmethynil. To address this disadvantage, they changed the substituent at each position. The patent was based on the indole structures with specific residues at positions 1, 3, and 5. The solubility and the lipophilicity were altered according to the locations or features of the substituents or the properties of the compound, such as the efficiency of inhibition of ICMT. Therefore, the team’s aim was to develop a series of compounds with the most effective ICMT inhibitory activity by adjusting the balance of hydrophilic and lipophilic properties of the indole-based compounds. Indoleamine-based analogs were synthesized by substituting various hydrophilic aromatic rings at position 5 to increase lipophilicity. In comparison with cysmethynil, which has lipophilicity of Log*D* 6.94 at pH 7.4 and solubility of 3.3 × 10^−7^ M, these new analogs (compound R 1-1–17; Fig. 5a) exhibited lower lipophilicities (Log*D*_7.4_ of 3.5–6.6) and higher solubility (8.7 × 10^−5^–1.8 × 10^−3^ M). To minimize the loss of energy from entropy when the side chain of indoleamines was flexibly transformed, they used tetrahydrocarbolines to restrict the conformational change of the diethylaminomethyl side chain in the indoleamines. Derivatives of tetrahydrocarbolines with various substituents at positions 1, 3, and 5 were also screened. The lipophilicities of these analogs have values ranging from Log*D*_7.4_ of 2.3–7.4 and solubility values from 8.8 × 10^−6^ to 1.9 × 10^−3^ M (compounds R2-1–11; Fig. 5b). Furthermore, the IC_50_ values of ICMT enzyme activity and anti-proliferative activity were determined using cancer cell lines. The compounds yielded IC_50_ values ranging from 0.8 to 10.3 μM for enzyme activity and from 2.1 to 14.7 and 2.01 to 17.4 μM for cell viability in MDA-MB-231 cells and PC3 cells, respectively. Among the analogs, compounds R1-11, 1-12, 1-14, 2-7, and 2-8 seem to be good candidates for anti-cancer drugs, because they have better lipophilicity, solubility, and anti-proliferative activity than standard compounds such as cysmethynil. According to the total evaluation considering lipophilicity, solubility, and anti-proliferative activity for an anti-cancer drug candidate, 1-11 is the most effective derivative for inhibition of ICMT. The presence of an *N*-octyl at position 1, which greatly increases the lipophilicity of the analog, is a shared feature with cysmethynil, implying that the balance between hydrophilicity and hydrophobicity is crucial. In contrast, 1-11 replaces a methyl phenyl group with a pyrimidine at position 5, thereby increasing the net solubility in water. In addition, this form of indoleamine was more effective in increasing the enzyme inhibitory activity of ICMT, the anti-proliferative effect, and solubility than were tetrahydrocarbolines. This result suggests that the conformational flexibility of the side chain is also a significant factor in the efficiency of ICMT inhibitors.

### Universidad Complutense De Madrid

**Fig. 6:**
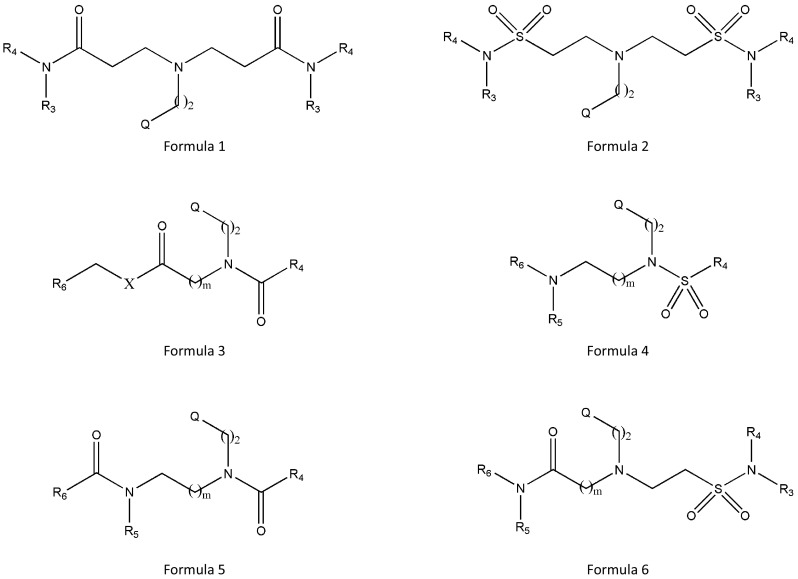
Formulas of ICMT inhibitors developed by Rodriguez (Universidad Complutense De Madrid)

Rodriguez Maria Luz Lopez’s group developed ICMT inhibitors as formulas 1, 2, 3, 4, 5, and 6. They produced a total of 22 inhibitors and confirmed the inhibitory activity of ICMT by enzyme assay and cytotoxicity by MTT assay using MCF-7 and MDA-MB-231 cells (Fig. 6). Among the 22 inhibitors, JAN showed a good balance between inhibition of ICMT activity and cell viability (ICMT activity inhibition = 71%; cytotoxicity of ICMT = 9.7 ± 0.1 μM for MCF-7 and 8.8 ± 0.3 μM for MDA-MB-231).

### Cancer Therapeutics CRC PTY Ltd

**Fig. 7:**
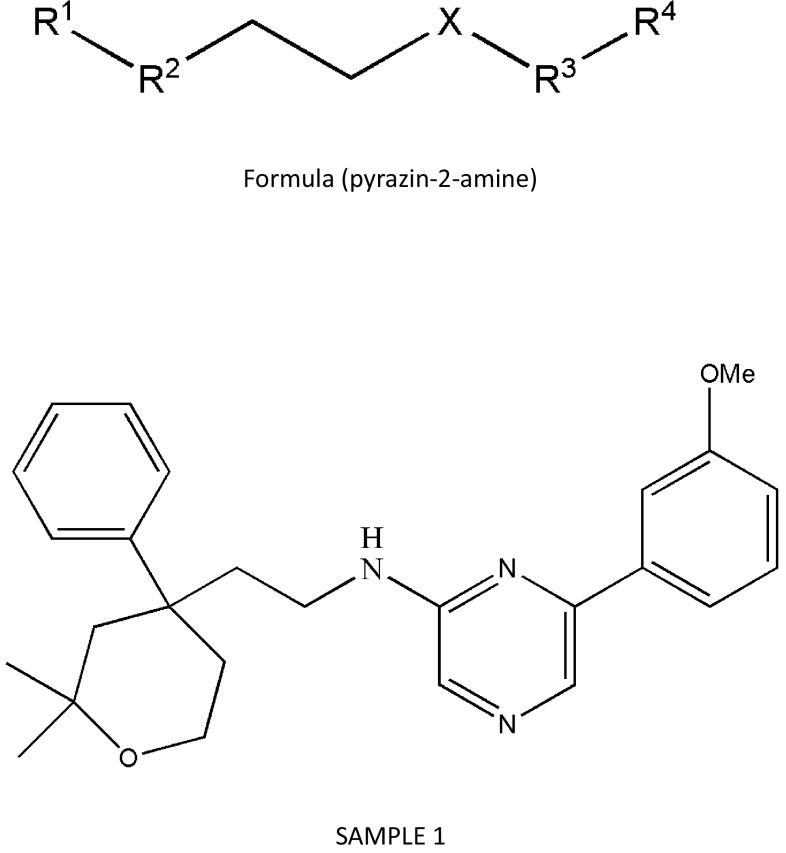
Pyrazin-2-amine formulas and ICMT inhibitor developed by Stevenson (Cancer Therapeutics CRC PTY Ltd)

Cancer Therapeutics CRC PTY Ltd has developed ICMT inhibitors using a formula based on pyrazin-2-amine (Fig. 7). These compounds have lower molecular weight than the other inhibitors described in this review. Of 31 compounds, C-2 has the lowest IC_50_ value (0.0014 μM) and has a chemical formula of A/-(2-(2,2-dimethyl-4-phenyl-tetrahydro-2H-pyran-4-yl)ethyl)-6-(3-methoxyphenyl)pyrazin-2-amine. Furthermore, the authors have mentioned that they are aiming to determine the pharmacological value of these compounds using in vitro and in vivo cancerous conditions.

## Discussion

The Ras protein family has been found to play a significant role in proliferative activity in many oncogenic cell lines. Mutations in Ras family members leading to their abnormal activation are common and are found in one-third of all human tumors. Therefore, there is a high demand for development of Ras inhibitors. Since the Ras protein undergoes a number of post-translational modifications during its “switching on” process, various types of inhibitors with distinct mechanisms of action are under development. Previously, farnesylation of Ras by FTase was the prime target in the Ras-mediated proliferative signaling pathway. Such FTase inhibitors (FTIs) showed promising inhibitory effects with low toxicity in mouse models; however, results of human clinical trials were rather disappointing. It is mostly agreed that the explanation for their poor inhibitory effect is “alternate prenylation”, in which the empty farnesylation site of Ras due to use of FTIs is geranylgeranylated by GGTase I. As a result, FTIs show limited inhibitory effects, because geranylgeranylated Ras maintains biological activity similar to farnesylated Ras, ultimately leading to oncogenic proliferation. In contrast, methylation of Ras by ICMT is both crucial and nonspecific to either type of prenylation. Because the geranylgeranylated Ras will still be affected by ICMT inhibitors, such inhibitors can ensure more effective inhibition of Ras-induced abnormal growth factor signaling in cancer cells compared with the previous approaches.

The previous studies also used different mechanisms of action to inhibit Ras methylation by ICMT. Introducing AdoHcy or its precursors diminishes Ras methylation; however, this mechanism is problematic, because it is not specific for ICMT but applies to all methylation. Another method is competitive inhibition of ICMT by structural analogs of the substrate prenylcysteine. AFC and AGGC mimic prenylated Ras and compete for the substrate access tunnel of ICMT. The third class of inhibitor was discovered when screening a diverse library of indole-based scaffolds. The selected inhibitors are small in size (~900 Da) and have greater permeability through cellular membranes than cysmethynil. This compound is a prototypical small-molecule ICMT inhibitor that was synthesized and patented at Duke University by Dr. Casey. This potent ICMT inhibitor forms a reversible complex (*K*_i_ = 2.4 μM) with ICMT and induces a conformational change toward a tighter complex (*K*_i_ = 0.1 μM). The final enzyme-inhibitor complex with a lower *K*_i_ shows decreased dissociation rates and augmented inhibitory effects from simple competitive inhibition, as seen in second-generation ICMT inhibitors. In other words, indole-based small-molecule inhibitors act as a competitive isoprenylated cysteine substrate as well as a non-competitive inhibitor of the methyl donor AdoMet.

Even though cysmethynil is a very potent ICMT inhibitor and a promising candidate for anti-cancer clinical trials, it was eliminated from drug development due to poor solubility in water. It barely complies with or violates the criteria set by Lipinski’s “Rule of Five” (RO5), including the number of rotatable bonds (*n* = 10) and high lipophilicity (Log*D*_7.4_ = 6.9). Overcoming these drawbacks might further enhance the effectiveness of the drug due to an improved drug delivery system (solubility) and increased specificity (fewer rotatable bonds). Two research groups at Singapore University made an attempt to enhance the drug. Various functional groups were substituted at positions 1, 3, and 5 of indole-based analogs, and the resultant compounds were screened as small-molecular ICMT inhibitors. Among the screened analogs, compound R1-11 (Fig. 5a) exhibited the most potent anti-proliferative activity in MDA-MB231 and PC3 cells, with the lowest IC_50_ values of 2.2 and 2.0 μM, respectively, compared with 26.8 ± 1.9 and 24.8 ± 1.5 μM for cysmethynil. Ramanujulu’s team pointed out that replacing acetamide at position 3 of cysmethynil with diethyl tertiary amine remarkably improved the anti-proliferative activity, although the enzyme activity of ICMT was not strongly increased, according to comparison of IC_50_ values. The ICMT IC_50_ value of compound R1-11 was low, at half that of cysmethynil, but other screened analogs with diethyl tertiary amine had IC_50_ values for ICMT that were about the same or three times higher. All the analogs exhibited noticeably improved anti-proliferative activities (IC_50 MDA-MB231_ = 2.6–14.8 μM). Since the diethyl tertiary amine of these analogs is nonpolar compared with the acetamide of cysmethynil, it appeared to contradict the findings for RO5, in which higher lipophilicity negatively influenced the effectiveness of the drug. Similar phenomena were observed when amides of cysmethynil were replaced by amines (compounds J5-1–10). Furthermore, the results from compounds J6-1–8 suggest that higher degrees of resonance capacity in R1 and lesser degrees of polarity in R2 contribute to the overall increased effectiveness. Therefore, an octyl side chain in position 1 (R2) of compound R1-11 appears to be the best choice. Although the researchers at Singapore University suggested the theory of balance between solubility and lipophilicity, it is reasonable to speculate that the R2 and R3 residues contribute to the stability of indole-based small-molecule inhibitors inside the substrate access tunnel of ICMT, thereby leading to lower EI* complex *K*_i_ values. Similarly, greater resonance capability at R1 might benefit from modulating the electron density of the molecule. Ramanujulu’s team also restricted flexibility by attaching tetrahydrocarbolines at positions 2 and 3 of cysmethynil, but the resulting compounds were not as potent as the initial indole form, indicating that a certain level of flexibility is beneficial. At Universidad Complutense De Madrid, inhibitors of ICMT were developed using formulas 1, 2, 3, 4, 5, and 6. However, the efficacy for ICMT enzyme activity and cytotoxicity were not superior to the previous inhibitors (Fig. 6). Cancer Therapeutics CRC PTY Ltd also developed ICMT inhibitors based on pyrazin-2-amine. Although their inhibitor has a very low IC_50_ value of 0.0014 μM, there are no data on solubility or ICMT enzyme activity; therefore, it is difficult to compare the efficacy (Fig. 7).

The compound R1-11 was determined to be the most potent indole-based small-molecule inhibitor. The prototypical small-molecule inhibitor cysmethynil still has reasonably good inhibitory effects on both ICMT and Ras-induced growth factor signaling despite its violation of RO5. Optimizing cysmethynil to RO5 compliance failed to improve its inhibitory activity; however, other analogs provided more insights into the inhibitor-ICMT interaction and new criteria for ICMT inhibitors that might supersede RO5 optimization. Since the use of ICMT inhibitors in the treatment of cancer is still at a preliminary stage with no therapeutically effective patented compounds in clinical trials, further investigations into the structural differences between cysmethynil and compound R1-11 could lead to development of a novel RO5-compliant small-molecular inhibitor that might advance future clinical fields. The inhibition of ICMT is an emerging and innovative area in the development of potential anti-cancer therapeutics. Since none of the ICMT inhibitors developed to date have been therapeutically effective in clinical trials, additional efforts in medicinal chemical and pharmacological fields will be further required to prepare a successful therapeutic model using ICMT inhibitors.
